# Neuroanatomical Abnormalities and Cognitive Impairments Are Shared by Adults with Attention-Deficit/Hyperactivity Disorder and Their Unaffected First-Degree Relatives

**DOI:** 10.1016/j.biopsych.2013.09.025

**Published:** 2014-10-15

**Authors:** Valentino Antonio Pironti, Meng-Chuan Lai, Ulrich Müller, Chris Martin Dodds, John Suckling, Edward Thomas Bullmore, Barbara Jacquelyn Sahakian

**Affiliations:** a Department of Psychiatry, University of Cambridge, Cambridge, United Kingdom; b Medical Research Council/Wellcome Trust Behavioural and Clinical Neuroscience Institute, University of Cambridge, Cambridge, United Kingdom; c Adult ADHD Clinic, Cambridgeshire & Peterborough National Health Service Foundation Trust, Cambridge, United Kingdom; d Autism Research Centre, Department of Psychiatry, University of Cambridge, Cambridge, United Kingdom; e Department of Psychiatry, College of Medicine, National Taiwan University, Taipei, Taiwan; f Department of Psychology, College of Life and Environmental Sciences, University of Exeter, Exeter, United Kingdom

**Keywords:** ADHD, biomarkers, endophenotypes, neuroimaging, neuropsychology, VBM

## Abstract

**Background:**

Attention-deficit/hyperactivity disorder (ADHD) is a highly heritable neurodevelopmental disorder, yet the search for genes with a definitive role in its etiology has been elusive. Deconstructing the disorder in its endophenotypic traits, where the variance is thought to be associated with a fewer number of genes, should boost the statistical power of molecular genetic studies and clarify the pathophysiology of ADHD. In this study, we tested for neuroanatomical and cognitive endophenotypes in a group of adults with ADHD, their unaffected first-degree relatives, and typically developing control subjects.

**Methods:**

Sixty participants, comprising 20 adults with ADHD, 20 unaffected first-degree relatives, and 20 typically developing control subjects matched for age and gender undertook structural magnetic resonance imaging scans. Voxel-based morphometry with DARTEL was performed to obtain regional gray and white matter volumes. General linear analyses of the volumes of brain regions, adjusting for age and total intracranial volume, were used to compare groups. Sustained attention and response inhibition were also investigated as cognitive endophenotypes.

**Results:**

Neuroanatomical abnormalities in gray matter volume in the right inferior frontal gyrus and white matter volume in the caudal portion of the right inferior fronto-occipital fasciculus were shared between ADHD probands and their unaffected first-degree relatives. In addition, impairments in sustained attention were also found to be shared between ADHD patients and their relatives.

**Conclusions:**

Cognitive impairments in sustained attention and neuroanatomical abnormalities in the right inferior frontal gyrus and the posterior part of right inferior fronto-occipital fasciculus are putative neurocognitive endophenotypes in adult ADHD.

Attention-deficit/hyperactivity disorder (ADHD) is a childhood-onset neurodevelopmental disorder (1), with symptoms persisting into adulthood in approximately 50% of individuals 2, 3. Several genetic studies have shown convincing evidence that ADHD is highly heritable 4, 5. Yet, attempts to find genes and the genomic variants that increase susceptibility to ADHD have led to inconsistent results (6). To increase statistical power to identify susceptibility genes in ADHD, endophenotype strategy has become appealing. Endophenotypes are internal, quantitative traits assumed to be closer to the expression of the genes than the clinical picture per se. If the clinical phenotype is reduced to its basic neurocognitive components, the number of genes associated with variation in these components might be fewer compared with the number of genes linked to the overt clinical phenotype. Reducing the number of genes to test for should enhance the statistical power in molecular genetic studies aimed at discovering susceptibility genes for a disorder 7, 8, 9, 10, 11. Brain morphology measured by magnetic resonance imaging (MRI) is likely to be a useful endophenotype because it meets several of the criteria an endophenotype should possess: heritable, associated with the disorder, and expressed at higher rates in unaffected relatives of probands with ADHD than individuals drawn from the general population 9, 11, 12. Durston *et al.* (13) measured brain morphology in children with ADHD and their unaffected siblings and found volume reduction in the right prefrontal cortex in both ADHD children and their siblings, demonstrating that neuroanatomical measurements may lead to discovering endophenotypes in childhood ADHD. Nonetheless, this is the only study on neuroanatomical endophenotypes in ADHD and only in children.

Indeed, there are more than two dozen structural MRI studies in children and only a few in adults (14). Overall, results show that the neuroanatomical impairments are located in neural networks subserving attention and executive functions such as dorsolateral prefrontal cortex, cerebellum, inferior parietal lobule, and anterior cingulate cortex, including white matter abnormalities in the cingulum bundle and the inferior and superior longitudinal fasciculi. Moreover, it is becoming apparent that neuroanatomical abnormalities at subcortical level generally identified in children might ameliorate with age 15, 16, 17, independent from medication status 16, 17. Recent reviews mainly based on works with children highlighted a brain network displaying functional and structural abnormalities in ADHD, the cingulo-frontal-parietal (CFP) cognitive/attention network 18, 19, 20, 21. The CFP network includes caudate nucleus and putamen at the subcortical level, and at the cortical level, it includes the cerebellum, dorsal anterior mid cingulate cortex, dorsolateral prefrontal cortex, ventrolateral prefrontal cortex including inferior frontal gyrus, and angular gyrus (19). These regions are thought to work in concert with each other to support normal cognition, attention, and motor control processes 18, 19.

The aim of this study was to test the hypothesis that adults with ADHD and their unaffected first-degree relatives share gray matter and white matter abnormalities using the CFP network as theoretical framework. Given the CFP network mainly includes cortical areas and that previous data showed the cortex as the primary source of volumetric abnormalities, particularly in adult ADHD 16, 22, gray matter volume analysis focused on a region of interest (ROI) including cortical areas and excluding subcortical regions. However, given the CFP network also includes part of the basal ganglia, we also focused our analysis on a region of interest including the caudate nucleus and putamen. For white matter, a whole-brain approach was adopted to explore both cortical and subcortical white matter differences. Given the CFP network is thought to underpin attentional and cognitive control, we also tested whether common cognitive dysfunctions seen in ADHD, such as response inhibition and sustained attention 23, 24, 25, are shared among adult ADHD probands and their unaffected first-degree relatives. To test these hypotheses, 20 adult ADHD probands, 20 unaffected first-degree relatives, and 20 typically developing control subjects underwent MRI scan and voxel based morphometry (VBM) analysis with diffeomorphic anatomic registration through an exponentiated lie algebra algorithm (DARTEL) and completed two computerized tasks tapping response inhibition and sustained attention.

## Methods and Materials

### Participants

Twenty ADHD patients, 20 unaffected first-degree relatives of ADHD patients, and 20 typically developing participants matched for age and gender were included. Written consent was obtained and the study was approved by the Cambridgeshire 3 Research Ethics Committee (REC: 09/H0306/38). Attention-deficit/hyperactivity disorder proband-relative pairs were recruited from the Adult ADHD Research Clinic, Addenbrooke’s Hospital, Department of Psychiatry, University of Cambridge. Patients received a diagnosis of ADHD according to DSM-IV Text Revision (26), based on a full clinical interview with the patient and an informant who had known the patient since childhood. The clinical assessment also included rating scales: Barkley Adult ADHD Rating Scale, self-report and informant report, childhood and adulthood symptoms (27), assessing childhood and adulthood symptoms from the perspective of the patient and the informant. Eligible patients were asked to contact a first-degree relative who undertook the same clinical protocol to screen for undiagnosed adult ADHD. Control participants were recruited via posters in the local community and completed the same screening procedure.

On the testing day, all participants were interviewed using the Mini International Neuropsychiatric Inventory (28) to screen for DSM-IV Axis I disorders and completed the Barkley Adult ADHD Rating Scale, self-report (29). Estimate of full IQ was obtained using the National Adult Reading Test (NART) (30). Neither control subjects nor first-degree relatives of ADHD probands showed ADHD symptoms meeting the DSM-IV Text Revision diagnostic threshold for ADHD. Moreover, they did not show clinically significant symptoms of another DSM-IV Text Revision disorder. Finally, ADHD participants did not show relevant symptoms of a comorbid disorder reaching clinical significance for a formal DSM-IV Text Revision diagnosis. To reduce confounds resulting from other major psychiatric and neurological conditions, exclusion criteria were 1) full IQ ≤ 90; 2) current or past history of pervasive developmental disorder, any neurological disorder (including tic disorders), bipolar disorder, substance-use disorders, schizophrenia, or other psychotic disorders; 3) current major depressive disorder; and 4) contraindications to a MRI scan. To minimize the impact of psychotropic medication on cognitive performance, participants were asked to omit taking those 24 hours before testing 31, 32 and were asked to refrain from consuming alcohol or caffeine-containing drinks on the day of testing. The ADHD group comprised 16 patients with combined type and 4 with inattentive type; 16 of them were medicated with methylphenidate, and 4 were not receiving medication for ADHD. None of the participants had a NART full IQ below 90.

### Neuropsychological Tasks

All participants completed the Cambridge Neuropsychological Test Automated Battery stop signal task of response inhibition (33) and Cambridge Neuropsychological Test Automated Battery rapid visual information processing test of sustained attention (RVP) (34) (see Supplement 1 for details).

### MRI Analysis

Images were acquired at Wolfson Brain Imaging Centre, University of Cambridge, using a Siemens TIM Trio 3T system (Siemens Medical Solutions, Erlangen, Germany) (see Supplement 1 for details). Voxel based morphometry analysis was performed using SPM8 (Welcome Department of Imaging Neuroscience, London, United Kingdom). To improve the registration of the MRI images, we used DARTEL (35). DARTEL provides improved registration accuracy compared with conventional VBM, and VBM with DARTEL is more sensitive than conventional VBM methods (36). The following processing steps were implemented: 1) structural images of the 60 participants were used to create a study-specific template; 2) structural images of each participant were applied to unified segmentation and initial import and the imported data were warped to the study-specific template; 3) Jacobian modulation was applied to preserve information about local volumes, generating standard-space modulated images; and 4) images were smoothed with an 8 mm full-width at half maximum kernel. Group level statistical analyses were performed with SPM8 using general linear model, with total intracranial volume (TIV) and age included as covariates.

Conjunction analysis testing for global differences at cluster level was used. Only clusters surviving at familywise error (FWE) or false discovery rate *p* < .05, corrected at cluster level with a conservative cluster-forming threshold, αc of .001, are reported. Conjunction analysis testing for global differences allows testing the null hypothesis of no differences between ADHD patients versus control subjects and first-degree relatives versus control subjects [for details about the method see 37, 38]. For gray matter, we were mainly interested in cortical areas comprised in the CFP cognitive/attention network; for this reason, one ROI was employed using WFU PickAtlas (ANSIR Laboratory, Department of Radiologic Sciences WFU School of Medicine, Winston-Salem, North Carolina) (39) that included frontal, parietal, temporal, occipital lobes, and cerebellum. A second ROI analysis focused on two regions in the basal ganglia (putamen and caudate nucleus) that are considered part of the CFP network.

Within these ROIs, conjunction analyses testing for global differences at the cluster level was conducted with the conjoined contrasts as (ADHD < control subjects: −1 0 1; relatives < control subjects: 0 −1 1) to identify decreased gray matter volume in the ADHD group and their unaffected relatives compared with control subjects. To identify increased gray matter volume in the ADHD group and their unaffected relatives compared with control subjects, the conjoined contrasts were (ADHD > control subjects: 1 0 −1; relatives > control subjects: 0 1 −1) [a similar approach is used in (40)].

For white matter analysis, to explore both cortical and subcortical abnormalities in a nonbiased manner, a whole-brain approach was used. The same conjunction analysis used for gray matter analysis testing for global differences at the cluster level was carried out with the conjoined contrasts as (ADHD < control subjects: −1 0 1; relatives < control subjects: 0 −1 1) to detect decreased white matter volume in the ADHD group and their unaffected relatives compared with control subjects. To identify increased white matter volume in the ADHD group and their unaffected relatives compared with control subjects, the conjoined contrasts were (ADHD > control subjects: 1 0 −1; relatives > control subjects: 0 1 −1).

To characterize differences between the three groups, cluster regional volume estimates surviving the conjunction analyses were extracted using MarsBaR (41) and imported into SPSS to perform analysis of variance ROI analyses with group (ADHD, relatives, control subjects) as a fixed factor and cluster regional volume estimates as dependent variables. This ROI analysis methodology has been widely implemented in other studies 42, 43. Given the number of comparisons, a Bonferroni correction was applied; however, when Levene’s test for equality of variance was significant, Tamhane correction was used. All brain coordinates are given in Montreal Neurological Institute (MNI) convention. The International Consortium of Brain Mapping Diffusion Tensor Imaging-81 atlas (International Consortium of Brain Mapping Diffusion Tensor Imaging workgroup) was used to assign anatomical labels to white matter structures. Bivariate Pearson correlation analysis between sustained attention performance, response inhibition, and volume estimates from significant clusters was calculated.

## Results

### Demographic and Clinical Characteristics

The three groups did not differ in age and gender. Attention-deficit/hyperactivity disorder group scored four points lower than typically developing control subjects on NART full IQ. Attention-deficit/hyperactivity disorder differed from relatives and control subjects in self-reported current and childhood symptoms. Unaffected first-degree relatives were significantly different from ADHD and control groups on self-reported current hyperactive/impulsive symptoms, childhood total symptoms, childhood hyperactive/impulsive, and childhood inattentive symptoms (Table 1).

**Table 1:** Sample Characteristics

	ADHD		Relatives		Control Subjects			
	Mean	SD	Mean	SD	Mean	SD	*F*_2,57_	*p* Value
Age	32.2	10.31	38.85	15.31	32.55	5.8	2.245	*p* = .115
Gender, % Female	15		50		35		5.550	*p* = .062^a^
NART Full IQ	115.26	6.15	116.59	5.28	119.49	3.27	3.679	*p* = .031^b^
BAARS Current Total Symptoms	36.15	12.39	10.00	7.38	5.20	4.29	73.5	*p* < .001^b^^c^
BAARS Current Hyperactive/Impulsive	18.40	6.98	5.25	4.13	2.50	2.50	60.11	*p* < .001^b^^c^^d^
BAARS Current Inattentive Symptoms	17.75	5.96	4.75	3.89	2.70	2.77	68.44	*p* < .001^b^^c^
BAARS Childhood Total Symptoms	41.35	11.93	14.20	10.28	4.85	6.65	73.78	*p* < .001^b^^c^^d^
BAARS Childhood Hyperactive/Impulsive	20.65	6.23	6.60	4.45	2.15	3.44	79.45	*p* < .001^b^^c^^d^
BAARS Childhood Inattentive Symptoms	20.70	6.04	7.60	6.23	2.70	4.07	56.57	*p* < .001^b^^c^^d^

Sample characteristics and clinical measures.

ADHD, attention-deficit/hyperactivity disorder; BAARS, Barkley Adult ADHD Rating Scale; NART, National Adult Reading Test.

a χ².

b The ADHD group differs significantly from the control subjects.

c The ADHD group differs significantly from the relatives.

d Relatives differ significantly from control subjects.

### Behavioral Analysis

Table 2 shows mean and standard deviation of the test scores for the three groups. There were not significant differences between the three groups for stop signal reaction time (SSRT) scores (*F*_2,55_ = 2.637, *p* = .081). Post hoc analysis showed a trend toward significance when comparing the ADHD group versus control subjects (*p* = .064). First-degree relatives were not different from the ADHD and control groups (*p* = .271; 1.000). Given the trend, we reran the analysis with a less stringent correction for multiple comparisons (Sidak), yet the significance remained unchanged.

**Table 2:** Response Inhibition and Sustained Attention Scores According to Group

	ADHD		Relatives		Control Subjects	
	Mean	SD	Mean	SD	Mean	SD
SSRT	187.56	76.52	158.06	33.40	147.02	43.02
RVP-Total Hits	15.95	5.22	15.35	5.67	22.00	3.57

Mean and standard deviation of the SST and RVP scores.

ADHD, attention-deficit/hyperactivity disorder; RVP, rapid visual information processing test of sustained attention; SSRT, stop signal reaction time; SST, stop signal task of response inhibition.

**Figure 1:**
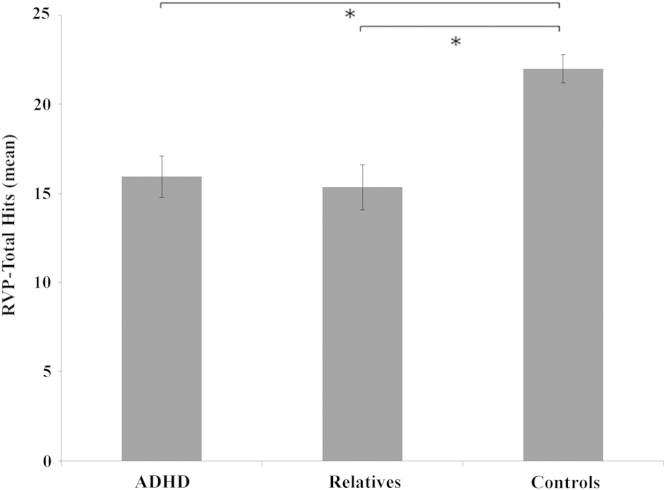
Sustained attention score according to group. Higher score indicates better performance. Attention-deficit/hyperactivity disorder (ADHD) and relatives did not differ from each other and were performing significantly worse than control subjects. *For significant differences, *p* < .05. Error bars represent standard error of the mean (SEM). RVP, rapid visual information processing test of sustained attention.

A main effect of group was found for the sustained attention score (RVP-total hits) (*F*_2,55_ = 6.058, *p* = .004). Post hoc analysis showed that the ADHD group performed worse than the control group (*p* = .025). First-degree relatives were not different from ADHD (*p* = 1.000) but performed significantly worse than the control group (*p* = .005) (Figure 1).

### Neuroimaging Analysis

Multivariate analysis of covariance assessing for TIV and total brain volume with group as a fixed factor and age as a covariate showed no differences between the three groups (TIV: *F*_2,56_ = 1.536, *p* = .224; total brain volume: *F*_2,56_ = 1.827, *p* = .170).

### Gray Matter Volume Analysis: Decrease in Gray Matter Volume

**Figure 2:**
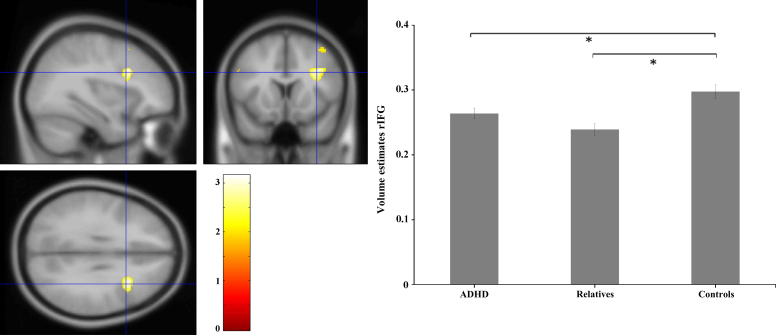
Significant cluster of decreased gray matter volume in the right inferior frontal gyrus (rIFG) in attention-deficit/hyperactivity disorder (ADHD) and first-degree relatives compared with control subjects. Peak cluster voxel located at Montreal Neurological Institute coordinates x = 34, y = 15, z = 30. On the right panel, mean volume estimates according to group. *For significant differences. Bars represent standard error of the mean (SEM).

Region of interest conjunction analysis showed a cluster of 448 voxels located at the right inferior frontal gyrus (rIFG) (cluster surviving at FWE *p* = .028; MNI x = 34 y = 15 z = 30) (Figure 2). To characterize these abnormalities, we extracted the volume estimates from this cluster and imported them into SPSS for an analysis of variance.

Results showed a main effect of group (*F*_2,57_ = 10.259, *p =* .0002). Post hoc comparisons revealed that the ADHD group had significantly decreased gray matter volume in the rIFG compared with control subjects (*p* = .035); first-degree relatives were not different from the ADHD probands (*p* = .184) but differed from the control group (*p* < .001), suggesting that abnormal decrease in gray matter volume in the rIFG was shared by both ADHD probands and their first-degree relatives. Region of interest conjunction analysis on the putamen and caudate nucleus did not show significant decrease in gray matter volume in these regions.

### Gray Matter Volume Analysis: Increase in Gray Matter Volume

**Figure 3:**
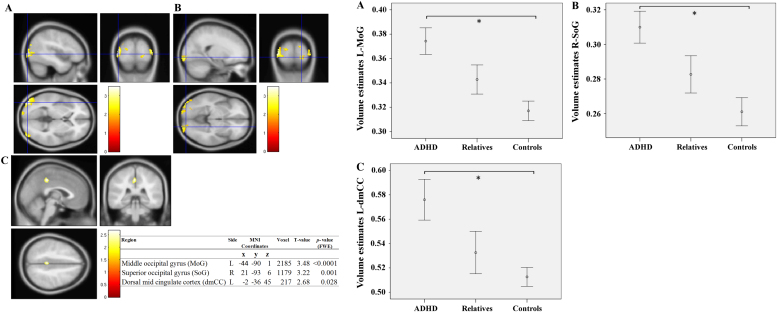
Significant clusters of increased gray matter volume in attention-deficit/hyperactivity disorder (ADHD) and first-degree relatives compared with control subjects. **(A)** Left middle occipital gyrus (L-MoG); **(B)** Right superior occipital gyrus (R-SoG); **(C)** Left dorsal mid cingulate cortex (L-dmCC). On the right panel, mean volume estimates according to group. *For significant differences. Bars represent standard error of the mean (SEM). L, left; MNI, Montreal Neurological Institute; R, right.

Region of interest conjunction analysis showed three significant clusters surviving cluster-level FWE correction at *p* < .05 (Figure 3). Two clusters were located in the occipital cortex, with peak voxels centered at the left middle occipital gyrus (L-MoG, Figure 3A) and at the right superior occipital gyrus (R-SoG, Figure 3B). One cluster was located in the posterior part of the left dorsal mid cingulate cortex (L-dmCC, Figure 3C).

Analysis of variance on the regional volume estimates extracted from the L-MoG showed a main effect of group (*F*_2,57_ = 7.576, *p =* .001). Post hoc comparisons revealed that the ADHD group had significantly increased gray matter volume in the L-MoG compared with control subjects (*p* = .001); first-degree relatives were not different from the ADHD probands (*p* = .110) or from the control group (*p =* .259). Analysis of variance on volume estimates extracted from R-SoG showed a main effect of group (*F*_2,57_ = 6.733, *p =* .002), and post hoc comparisons revealed that the ADHD group had significantly increased gray matter volume in the R-SoG compared with control subjects (*p* = .002); first-degree relatives were not different from the ADHD probands (*p* = .137) or from the control group (*p =* .334). Similar results were found in the L-dmCC showing a main effect of group (*F*_2,57_ = 4.904, *p =* .011), and post hoc comparisons revealed that the ADHD group had significantly increased gray matter volume in the L-dmCC compared with control subjects (*p* = .010); first-degree relatives were not different from the ADHD probands (*p* = .121) or from the control group (*p =* 1.000). Region of interest conjunction analysis on the putamen and caudate nucleus did not reveal significant increase in gray matter volume.

### White Matter Volume Analysis: Decrease in White Matter Volume

Whole-brain conjunction analysis did not reveal significant decrease in white matter volume between groups.

### White Matter Volume Analysis: Increase in White Matter Volume

**Figure 4:**
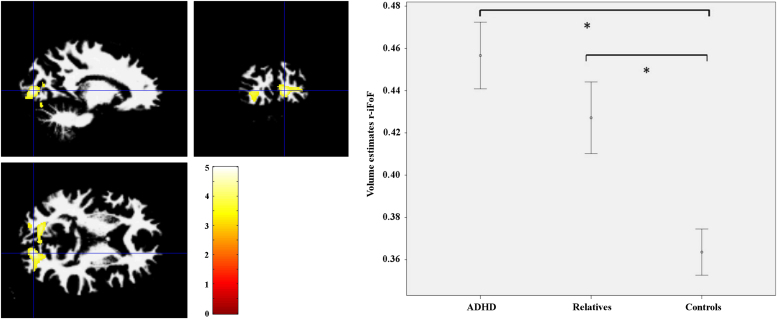
Blue cross highlights the cluster local maxima on the posterior portion of the right inferior fronto-occipital fasciculus (r-iFoF) where white matter volume is increased when comparing attention-deficit/hyperactivity disorder (ADHD) and first-degree relatives with control subjects (Montreal Neurological Institute coordinates x = 15, y = −90, z = 4; cluster level correction, familywise error *p* < .05). Right panel shows mean volume estimates extracted from this cluster of 288 voxels, according to group). *For significant differences. Bars represent standard error of the mean.

Whole-brain conjunction analysis showed a cluster of 288 voxels with peak local maxima (MNI coordinates x = 15, y = −90, *z* = 4) at the posterior portion of the right inferior fronto-occipital fasciculus (r-iFoF) (cluster-level FWE correction *p* < .05) (Figure 4). Analysis of variance showed a main effect of group (*F*_2,57_ = 10.337, *p* < .001). Post hoc analysis confirmed that the ADHD group had an abnormal increase in white matter volume compared with control subjects (*p* < .001) and that there were no differences between the ADHD group and their first- degree relatives (*p* = .343); first-degree relatives were statistically different from control subjects (*p* = .011). The results suggest that white matter abnormalities in the posterior portion of the r-iFoF were shared between ADHD probands and their unaffected first-degree relatives.

### Correlation between Neuroanatomical and Cognitive Measures

**Figure 5:**
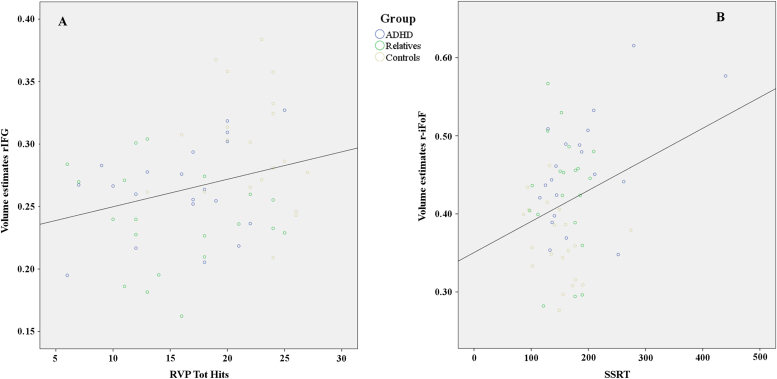
**(A)** Scatterplot showing that participants with greater gray matter volume in the right inferior frontal gyrus (rIFG) had better sustained attention performance on the rapid visual information processing test of sustained attention (RVP) task (correlation coefficient, *r* = .267; linear correlation constant, *R*^*2*^ = .071, *p* = .039). **(B)** Scatterplot showing that participants with greater white matter volume in the posterior portion of the right inferior fronto-occipital fasciculus (r-iFoF) had worse response inhibition performances (longer stop signal reaction time [SSRT]) on the stop signal task (the correlation coefficient, *r* = .294; the linear correlation constant, *R*^*2*^ = .086, *p* = .023). ADHD, attention-deficit/hyperactivity disorder; Tot, Total.

Bivariate Pearson correlation was calculated between gray and white matter volume estimates (extracted from the rIFG and r-iFoF) and sustained attention and response inhibition scores (RVP-total hits and SSRT, respectively) across groups. Gray matter volume estimates in the rIFG correlated with RVP-total hits, but not with SSRT, while white matter volume estimates in the r-iFoF correlated with SSRT but not with sustained attention score (Table 3; Figure 5).

**Table 3:** Pearson Correlations between Sustained Attention Performance, Response Inhibition, and Volume Estimates Extracted from the Right Inferior Frontal Gyrus and the Posterior Portion of Right Inferior Fronto-occipital Fasciculus

		RVP-Total Hits	SSRT
r-IFG	Pearson	.267^a^	.002
	*p* value	.039	.985
r-iFoF	Pearson	−.103	.294^a^
	*p* value	.435	.023

Significant correlation between gray and white matter volume and measure of sustained attention and response inhibition.

r-IFG, right inferior frontal gyrus; r-iFoF, right inferior fronto-occipital fasciculus; RVP, rapid visual information processing test of sustained attention; SSRT, stop signal reaction time.

a Significant correlation (two tails).

## Discussion

The aim of this study was to test for putative neuroanatomical and cognitive endophenotypes in adult ADHD in the CFP cognitive/attention network, which is thought to have a role in the pathophysiology of ADHD. Results could not be explained by nonspecific confounds such as age or variation in total intracranial or brain volume, since these were not statistically different between groups. Moreover, the effects of age and TIV were controlled for in the analyses. Although the ADHD group had a lower full IQ compared with control subjects, the first-degree relatives of ADHD participants had an IQ comparable with the control and the ADHD groups. Therefore, shared impairments between relatives and ADHD probands are less likely to be explained in full by variability in intellectual abilities. Finally, despite not being statistically significant, there were trend-level gender differences across groups. However, previous research in adult ADHD has shown no evidence that gender moderates the phenotypic expression of the disorder or the patterns of cognitive and psychosocial functioning 44, 45.

Here, we identified both neuroanatomical and cognitive putative endophenotypes. Neuroanatomically, the ADHD group had gray matter volume decrease in the rIFG and white matter volume increase in the posterior part of the r-iFoF, and these abnormalities were shared by both ADHD probands and their unaffected first-degree relatives. Moreover, sustained attention impairments were also shared among ADHD probands and their unaffected first-degree relatives. This suggests that these abnormalities are at least partly mediated by factors common to both groups and might indicate vulnerability markers for adult ADHD. We did not test for heritability of sustained attention, which is another criteria to be met for a putative endophenotype; however, others have shown that sustained attention is substantially genetically determined, with heritability estimates of about 60% (46). Our results demonstrate that first-degree relatives of ADHD probands, while not behaviorally expressing the disorder, have similar neurocognitive deficits as their ADHD relatives.

Decrease in gray matter density in the rIFG in ADHD has been observed in other studies (47). Interestingly, activation in the rIFG is not uniquely related to response inhibition but instead to attentional allocation when a stimulus is detected. Specifically, the rIFG is recruited when important cues are detected, regardless of whether the detection is followed by inhibition of a motor response (48). The present correlation result is consistent with findings from Hampshire *et al*. (48). They found a positive correlation between gray matter volume in the rIFG and sustained attention scores, such that the less gray matter volume, the poorer the performance on a task specifically requiring vigilance and attentional allocation. These results suggest that impairments in sustained attention seen in ADHD and first-degree relatives might be, in part, related to rIFG, since gray matter abnormalities were also shared among our group of adults with ADHD and their unaffected first-degree relatives.

A trend toward significant impairments in response inhibition in the ADHD group was present, and therefore we cannot exclude that with an increase in sample size results would become significant. However, the absence of clear impairments, even when using a less stringent correction for multiple comparisons, and the lack of correlation between response inhibition measure and rIFG volume are consistent with previous research showing that in individuals with ADHD impulsivity improves with age, while attentional impairments remain (49). White matter abnormalities in the posterior part of the r-iFoF were shared between the ADHD group and the first-degree relatives, suggesting that white matter abnormalities in this portion of the connectome are an endophenotype for adult ADHD. Importantly, this result is consistent with previous research showing white matter volume increase in adult ADHD (22). We did not test for heritability; however, previous studies have shown that one third of the variance of the inferior fronto-occipital fasciculus (iFoF) is genetically driven (50). Functionally, the integrity of the iFoF correlates with performance on executive function tasks (51). The present positive correlation between white matter volume in the iFoF and SSRT score might suggest that an abnormal increase in white matter volume in the iFoF reduces response inhibition abilities; however, this correlation becomes a trend without all data points included, and therefore this finding requires further replication. Nonetheless, this result is consistent with recent research highlighting a role of the iFoF in stopping performance (52).

It is known that white matter continues to develop until the late 20s (53); thus, it is plausible that some genetic processes mediating white matter development may be altered in adult ADHD, as well as in their unaffected first-degree relatives. Our results are consistent with the view that neurotrophic factors, which support neuronal survival and differentiation during development and participate in synaptic efficiency and neuronal plasticity in the adult nervous system, might be candidates in the pathophysiology of ADHD. There are known genes, such as the *NTRK1* and *NTRK2*, that encode a high-affinity receptor for nerve growth factor, a neurotrophin involved in neural development and myelination. A variant in the neurotrophin gene, BDNF, has previously been associated with white matter integrity in the iFoF in young adults (54). Given that we found white matter abnormalities in the r-iFoF, it is intriguing to hypothesize a potential role of this gene variant in the pathophysiology of ADHD. Moreover, given the link between r-iFoF and stopping performances, it might be that the same gene also has an effect on response inhibition variability. Research has already identified other potential associations between adult ADHD and other nerve growth factor receptors such as ciliary neurotrophic factor receptor and has suggested association of NTRK2 in childhood ADHD (55). Using endophenotypes identified here, further research might explore the role of NTRK1, NTRK2, and BDNF genes in adult ADHD.

Attention-deficit/hyperactivity disorder individuals had abnormal increase in gray matter volume compared with control subjects in the L-MoG, R-SoC, and L-dmCC. Recently, Seidman *et al.* (56) found gray matter volume increase in occipital regions in adult ADHD. Occipital cortex interacts with the dorsal attentional network to maintain attention and to suppress attention to irrelevant stimuli (57). Distractibility, which can manifest as a failure to ignore extraneous stimuli, is one of the core symptoms of ADHD. Therefore, abnormalities in the occipital cortex might relate to impairments in early-stage attentional mechanisms (58). We also reported an increase in gray matter volume in the posterior part of the middle cingulate cortex. Although increase in gray matter volume is rarely emphasized in the literature on ADHD, our results are consistent with a recent meta-analysis examining more than 300 participants with ADHD (16). From a functional standpoint, the posterior part of the dorsal mid cingulate cortex is part of the default network that is considered a physiological baseline of the brain where activity is consistently diminished during cognitive tasks and increased during rest (59). Intriguingly, some evidence suggests that ADHD might be also linked to dysfunction in the default mode network (60), with abnormal recruiting of the middle cingulate cortex in adults with ADHD seen as a source of poor attentional performance during the RVP task (61). Increase in white and gray matter volume might relate to abnormalities in the typical neuroanatomical developmental trajectories (62), which, in turn, might mediate overt behavior in adults with ADHD. These results may be the consequence of a relative imbalance in gray-white matter ratio during development in which there is excessive growth or, more likely, ineffective pruning. Research has shown that cortical pruning in ADHD is not as effective as in typically developing individuals (63). In addition, the present results are consistent with findings showing that slow rate of cortical thinning (reflecting cortical pruning) correlates with hyperactivity/impulsivity in children, such that individuals with the more symptoms show slower rate of cortical thinning (64).

In summary, we have demonstrated for the first time that neuroanatomical and cognitive impairments implicated in ADHD in adulthood are shared between individuals with ADHD and their unaffected first-degree relatives, highlighting neurocognitive vulnerability markers for adult ADHD. These findings correspond with the recent development of the Research Domain Criteria approach (65) and might have implications for facilitating treatment discovery tuned by a biomarker approach (66).

## References

[1] Kieling C., Kieling R.R., Rohde L.A., Frick P.J., Moffitt T., Nigg J.T. (2010). The age at onset of attention deficit hyperactivity disorder. Am J Psychiatry.

[2] Faraone S.V. (2007). ADHD in adults--a familiar disease with unfamiliar challenges. CNS Spectr.

[3] Wilens E., Faraone S.V., Biederman J. (2004). Attention-deficit/hyperactivity disorder in adults. JAMA.

[4] Castellanos F.X., Tannock R. (2002). Neuroscience of attention-deficit/hyperactivity disorder: The search for endophenotypes. Nat Rev Neurosci.

[5] Franke B., Faraone S.V., Asherson P., Buitelaar J., Bau C.H., Ramos-Quiroga J.A. (2011). The genetics of attention deficit/hyperactivity disorder in adults, a review. Mol Psychiatry.

[6] Hinney A., Scherag A., Jarick I., Albayrak O., Putter C., Pechlivanis S. (2011). Genome-wide association study in German patients with attention deficit/hyperactivity disorder. Am J Med Genet B Neuropsychiatr Genet.

[7] Doyle A.E., Willcutt E.G., Seidman L.J., Biederman J., Chouinard V.A., Silva J., Faraone S.V. (2005). Attention-deficit/hyperactivity disorder endophenotypes. Biol Psychiatry.

[8] Aron A.R., Poldrack R.A. (2005). The cognitive neuroscience of response inhibition: Relevance for genetic research in attention-deficit/hyperactivity disorder. Biol Psychiatry.

[9] Almasy L., Blangero J. (2001). Endophenotypes as quantitative risk factors for psychiatric disease: Rationale and study design. Am J Med Genet.

[10] Leboyer M., Leboyer M., Bellivier F., Jouvent R., Nosten-Bertrand M., Mallet J. (1998). Psychiatric genetics: Search for phenotypes. Trends Neurosci.

[11] Gottesman I.I., Gould T.D. (2003). The endophenotype concept in psychiatry: Etymology and strategic intentions. Am J Psychiatry.

[12] Waldman I.D., Rowe D.C., Abramowitz A., Kozel S.T., Mohr J.H., Sherman S.L. (1998). Association and linkage of the dopamine transporter gene and attention-deficit hyperactivity disorder in children: Heterogeneity owing to diagnostic subtype and severity. Am J Hum Genet.

[13] Durston S., Hulshoff Pol H.E., Schnack H.G., Buitelaar J.K., Steenhuis M.P., Minderaa R.B. (2004). Magnetic resonance imaging of boys with attention-deficit/hyperactivity disorder and their unaffected siblings. J Am Acad Child Adolesc Psychiatry.

[14] Seidman L.J., Valera E.M., Makris N. (2005). Structural brain imaging of attention-deficit/hyperactivity disorder. Biol Psychiatry.

[15] Frodl T., Skokauskas N. (2012). Meta-analysis of structural MRI studies in children and adults with attention deficit hyperactivity disorder indicates treatment effects. Acta Psychiatr Scand.

[16] Nakao T., Radua J., Rubia K., Mataix-Cols D. (2011). Gray matter volume abnormalities in ADHD: Voxel-based meta-analysis exploring the effects of age and stimulant medication. Am J Psychiatry.

[17] Castellanos F.X., Lee P.P., Sharp W., Jeffries N.O., Greenstein D.K., Clasen L.S. (2002). Developmental trajectories of brain volume abnormalities in children and adolescents with attention-deficit/hyperactivity disorder. JAMA.

[18] Bush G. (2011). Cingulate, frontal, and parietal cortical dysfunction in attention-deficit/hyperactivity disorder. Biol Psychiatry.

[19] Bush G. (2010). Attention-deficit/hyperactivity disorder and attention networks. Neuropsychopharmacology.

[20] Hart H., Radua J., Nakao T., Mataix-Cols D., Rubia K. (2013). Meta-analysis of functional magnetic resonance imaging studies of inhibition and attention in attention-deficit/hyperactivity disorder: Exploring task-specific, stimulant medication, and age effects. JAMA Psychiatry.

[21] Cortese S., Kelly C., Chabernaud C., Proal E., Di Martino A., Milham M.P., Castellanos F.X. (2012). Toward systems neuroscience of ADHD: A meta-analysis of 55 fMRI studies. Am J Psychiatry.

[22] Seidman L.J., Valera E.M., Makris N., Monuteaux M.C., Boriel D.L., Kelkar K. (2006). Dorsolateral prefrontal and anterior cingulate cortex volumetric abnormalities in adults with attention-deficit/hyperactivity disorder identified by magnetic resonance imaging. Biol Psychiatry.

[23] Willcutt E.G., Doyle A.E., Nigg J.T., Faraone S.V., Pennington B.F. (2005). Validity of the executive function theory of attention-deficit/hyperactivity disorder: A meta-analytic review. Biol Psychiatry.

[24] Seidman L.J., Doyle A., Fried R., Valera E., Crum K., Matthews L. (2004). Neuropsychological function in adults with attention-deficit/hyperactivity disorder. Psychiatr Clin North Am.

[25] Chamberlain S.R., Robbins T.W., Winder-Rhodes S., Muller U., Sahakian B.J., Blackwell A.D., Barnett J. (2011). Translational approaches to frontostriatal dysfunction in attention-deficit/hyperactivity disorder using a computerized neuropsychological battery. Biol Psychiatry.

[26] American Psychiatric Association (2000).

[27] Barkley R. (2011).

[28] Sheehan D.V., Lecrubier Y., Sheehan K.H., Amorim P., Janavs J., Weiller E. (1998). The Mini-International Neuropsychiatric Interview (M.I.N.I.): The development and validation of a structured diagnostic psychiatric interview for DSM-IV and ICD-10. J Clin Psychiatry.

[29] Barkley R., Murphy K.R. (2005).

[30] Nelson H.E., O’Connell A. (1978). Dementia: The estimation of premorbid intelligence levels using the New Adult Reading Test. Cortex.

[31] Gualtieri C.T., Wargin W., Kanoy R., Patrick K., Shen C.D., Youngblood W. (1982). Clinical studies of methylphenidate serum levels in children and adults. J Am Acad Child Psychiatry.

[32] Turner D.C., Blackwell A.D., Dowson J.H., McLean A., Sahakian B.J. (2005). Neurocognitive effects of methylphenidate in adult attention-deficit/hyperactivity disorder. Psychopharmacology (Berl).

[33] Aron A.R., Fletcher P.C., Bullmore E.T., Sahakian B.J., Robbins T.W. (2003). Stop-signal inhibition disrupted by damage to right inferior frontal gyrus in humans. Nat Neurosci.

[34] Sahakian B.J., Owen A.M. (1992). Computerized assessment in neuropsychiatry using CANTAB: Discussion paper. J R Soc Med.

[35] Ashburner J. (2007). A fast diffeomorphic image registration algorithm. Neuroimage.

[36] Klein A., Andersson J., Ardekani B.A., Ashburner J., Avants B., Chiang M.C. (2009). Evaluation of 14 nonlinear deformation algorithms applied to human brain MRI registration. Neuroimage.

[37] Nichols T., Brett M., Andersson J., Wager T., Poline J.B. (2005). Valid conjunction inference with the minimum statistic. Neuroimage.

[38] Friston K.J., Holmes A.P., Price C.J., Buchel C., Worsley K.J. (1999). Multisubject fMRI studies and conjunction analyses. Neuroimage.

[39] Maldjian J.A., Laurienti P.J., Kraft R.A., Burdette J.H. (2003). An automated method for neuroanatomic and cytoarchitectonic atlas-based interrogation of fMRI data sets. Neuroimage.

[40] Belton E., Salmond C.H., Watkins K.E., Vargha-Khadem F., Gadian D.G. (2003). Bilateral brain abnormalities associated with dominantly inherited verbal and orofacial dyspraxia. Hum Brain Mapp.

[41] Brett M, Anton J, Valabregue R, Poline J (2002): Region of interest analysis using an SPM toolbox [abstract]. Presented at the 8th International Conference on Functional Mapping of the Human Brain, June 2–6, 2002, Sendai, Japan.

[42] Carmona S., Hoekzema E., Ramos-Quiroga J.A., Richarte V., Canals C., Bosch R. (2012). Response inhibition and reward anticipation in medication-naive adults with attention-deficit/hyperactivity disorder: A within-subject case-control neuroimaging study. Hum Brain Mapp.

[43] Egner T., Hirsch J. (2005). Cognitive control mechanisms resolve conflict through cortical amplification of task-relevant information. Nat Neurosci.

[44] Biederman J., Faraone S.V., Monuteaux M.C., Bober M., Cadogen E. (2004). Gender effects on attention-deficit/hyperactivity disorder in adults, revisited. Biol Psychiatry.

[45] Biederman J., Faraone S.V., Spencer T., Wilens T., Mick E., Lapey K.A. (1994). Gender differences in a sample of adults with attention deficit hyperactivity disorder. Psychiatry Res.

[46] Polderman T.J., Gosso M.F., Posthuma D., Van Beijsterveldt T.C., Heutink P., Verhulst F.C., Boomsma D.I. (2006). A longitudinal twin study on IQ, executive functioning, and attention problems during childhood and early adolescence. Acta Neurol Belg.

[47] Depue B.E., Burgess G.C., Bidwell L.C., Willcutt E.G., Banich M.T. (2010). Behavioral performance predicts grey matter reductions in the right inferior frontal gyrus in young adults with combined type ADHD. Psychiatry Res.

[48] Hampshire A., Chamberlain S.R., Monti M.M., Duncan J., Owen A.M. (2010). The role of the right inferior frontal gyrus: Inhibition and attentional control. Neuroimage.

[49] Spencer T.J., Biederman J., Mick E. (2007). Attention-deficit/hyperactivity disorder: Diagnosis, lifespan, comorbidities, and neurobiology. J Pediatr Psychol.

[50] Jahanshad N., Lee A.D., Barysheva M., McMahon K.L., de Zubicaray G.I., Martin N.G. (2010). Genetic influences on brain asymmetry: A DTI study of 374 twins and siblings. Neuroimage.

[51] Takeuchi H., Taki Y., Sassa Y., Hashizume H., Sekiguchi A., Fukushima A., Kawashima R. (2013). Brain structures associated with executive functions during everyday events in a non-clinical sample. Brain Struct Funct.

[52] Coxon J.P., Van Impe A., Wenderoth N., Swinnen S.P. (2012). Aging and inhibitory control of action: Cortico-subthalamic connection strength predicts stopping performance. J Neurosci.

[53] Sowell E.R., Peterson B.S., Thompson P.M., Welcome S.E., Henkenius A.L., Toga A.W. (2003). Mapping cortical change across the human life span. Nat Neurosci.

[54] Braskie M.N., Jahanshad N., Stein J.L., Barysheva M., Johnson K., McMahon K.L. (2012). Relationship of a variant in the NTRK1 gene to white matter microstructure in young adults. J Neurosci.

[55] Ribases M., Hervas A., Ramos-Quiroga J.A., Bosch R., Bielsa A., Gastaminza X. (2008). Association study of 10 genes encoding neurotrophic factors and their receptors in adult and child attention-deficit/hyperactivity disorder. Biol Psychiatry.

[56] Seidman L.J., Biederman J., Liang L., Valera E.M., Monuteaux M.C., Brown A. (2011). Gray matter alterations in adults with attention-deficit/hyperactivity disorder identified by voxel based morphometry. Biol Psychiatry.

[57] Castellanos F.X., Proal E. (2012). Large-scale brain systems in ADHD: Beyond the prefrontal-striatal model. Trends Cogn Sci.

[58] Ahrendts J., Rusch N., Wilke M., Philipsen A., Eickhoff S.B., Glauche V. (2011). Visual cortex abnormalities in adults with ADHD: A structural MRI study. World J Biol Psychiatry.

[59] Raichle M.E., MacLeod A.M., Snyder A.Z., Powers W.J., Gusnard D.A., Shulman G.L. (2001). A default mode of brain function. Proc Natl Acad Sci U S A.

[60] Sonuga-Barke E.J., Castellanos F.X. (2007). Spontaneous attentional fluctuations in impaired states and pathological conditions: A neurobiological hypothesis. Neurosci Biobehav Rev.

[61] Lawrence N.S., Ross T.J., Hoffmann R., Garavan H., Stein E.A. (2003). Multiple neuronal networks mediate sustained attention. J Cogn Neurosci.

[62] Giedd J.N., Lenroot R.K., Shaw P., Lalonde F., Celano M., White S. (2008). Trajectories of anatomic brain development as a phenotype. Novartis Found Symp.

[63] Duerden E.G., Tannock R., Dockstader C. (2012). Altered cortical morphology in sensorimotor processing regions in adolescents and adults with attention-deficit/hyperactivity disorder. Brain Res.

[64] Shaw P., Gilliam M., Liverpool M., Weddle C., Malek M., Sharp W. (2011). Cortical development in typically developing children with symptoms of hyperactivity and impulsivity: Support for a dimensional view of attention deficit hyperactivity disorder. Am J Psychiatry.

[65] Cuthbert B.N., Insel T.R. (2013). Toward the future of psychiatric diagnosis: The seven pillars of RDoC. BMC Med.

[66] Insel T.R., Voon V., Nye J.S., Brown V.J., Altevogt B.M., Bullmore E.T. (2013). Innovative solutions to novel drug development in mental health [published online ahead of print April 3]. Neurosci Biobehav Rev..

