# Supplementary material for: Neuroanatomical Abnormalities and Cognitive Impairments Are Shared by Adults with Attention-Deficit/Hyperactivity Disorder and Their Unaffected First-Degree Relatives
Source: Biol Psychiatry. 2014 Oct 15;76(8):639–47. doi: 10.1016/j.biopsych.2013.09.025 (PMC4183379; doi:10.1016/j.biopsych.2013.09.025)
Supplement: Supplementary file 1 — Supplementary Material [file mmc1.pdf]

Neuroanatomical Abnormalities and Cognitive Impairments are Shared by Adults with Attention-Deficit/Hyperactivity Disorder and Their Unaffected First Degree Relatives

Supplemental Information

Supplementary Methods & Materials

Neuropsychological Tasks And Behavioral Analysis

The Stop Signal Task measures the ability to inhibit a pre-potent motor response (stop signal reaction time - SSRT). Participants watched a computer screen on which a series of 320 arrows divided in five blocks were presented. Arrows randomly pointed either to the right (50%) or to the left (50%), and participants responded accordingly by pressing the appropriate button. On 25% of trials, a stop-signal (a beep) was heard and subjects had to inhibit their motor response. Linking the stop-signal delay and the inter-stimulus interval to the participant's performance, participants were able to successfully inhibit their responses to 50% of the stop trials. From these outcome measures the SSRT for each participant was computed (1).

The Rapid Visual information Processing test (RVP) measures visual sustained attention using numbers as stimuli. Participants watched a computer screen where pseudo-random digits (2 to 9) appeared one at a time at a frequency of 100 per minute. Participants would press a response button any time they detected one of three digit target sequences. The main outcome was the number of total correct responses (RVP-Total Hits). Multivariate analysis of covariance was used with group (ADHD, Relatives, Controls) as a fixed factor and SSRT and RVP-Total Hits as dependent variables. Age and National Adult Reading Test full IQ were included as covariates. Given the number of comparisons, a Bonferroni correction was applied; however,

when Levene's test for equality of variance was significant, Tamhane correction was used. SPSS version 21 (http://www.spss.com/) was used for all behavioral analysis.

Magnetic Resonance Imaging Acquisition

T1-weighted magnetic resonance scans were acquired using a magnetization-prepared rapid acquisition gradient-echo sequence (176 slices, 1 mm thickness, TR = 2300 ms, TE = 2.98 ms, TI = 900 ms, flip angle = 9°, field of view = 240 x 256) and manually aligned to the anterior commissure – posterior commissure line. Voxel based morphometry analysis was performed using SPM8 (Welcome Department of Imaging Neuroscience, London).

Supplemental References

1. Aron AR, Fletcher PC, Bullmore ET, Sahakian BJ, Robbins TW (2003): Stop-signal inhibition disrupted by damage to right inferior frontal gyrus in humans. *Nat Neurosci*. 6:115-116.
